# Evaluation of the necessity for chest drain placement following thoracoscopic wedge resection

**DOI:** 10.1007/s00595-016-1414-5

**Published:** 2016-09-29

**Authors:** Ting-Yu Lu, Jian-Xun Chen, Pin-Ru Chen, Yu-Sen Lin, Chien-Kuang Chen, Pei-Yu Kao, Tzu-Ming Huang, Hsin-Yuan Fang

**Affiliations:** Division of Thoracic Surgery, Department of Surgery, China Medical University Hospital, No. 2, Yude Rd., North Dist., 404 Taichung City, Taiwan

**Keywords:** Chest tubes, Drainage, Pulmonary surgical procedure, Video-assisted thoracoscopic surgery, Pulmonary surgical procedure

## Abstract

**Purpose:**

To evaluate the outcomes of patients who underwent thoracoscopic wedge resection without chest drain placement.

**Methods:**

The subjects of this retrospective study were 89 patients, who underwent thoracoscopic wedge resection at our hospital between January, 2013 and July, 2015. A total of 45 patients whose underlying condition did not meet the following criteria were assigned to the “chest drain placement group” (group A): peripheral lesions, healthy lung parenchyma, no intraoperative air leaks, hemorrhage or effusion accumulation, and no pleural adhesion. The other 44 patients whose underlying condition met the criteria were assigned to the “no chest drain placement group” (group B). Patient characteristics, specimen data, and postoperative conditions were analyzed and compared between the groups.

**Results:**

Group A patients had poorer forced expiratory volume in one second (FEV1) values, less normal spirometric results, significantly higher resected lung volume, a greater maximum tumor-pleura distance, and a larger maximum tumor size. They also had a longer postoperative hospital stay. There was no difference between the two groups in postoperative complications.

**Conclusions:**

Avoiding chest drain placement after a thoracoscopic wedge resection appears to be safe and beneficial for patients who have small peripheral lesions and healthy lung parenchyma.

## Introduction

Traditionally, a chest drain is left in the pleural cavity after thoracoscopic wedge resection of the lung to mitigate against possible hemorrhage and air or lymphatic leakage [1, 2]. However, placing a chest drain is associated with pain, immobilization, an increased risk of wound infection, and poor healing. These adverse effects may cause postoperative complications and prolong the hospital stay [3–6]. Minimally invasive thoracoscopic wedge resection of the lung has been performed routinely at our institution over the last 5 years. Unless a patient had emphysematous changes of the lung or an incorrect staple application during a lung parenchyma resection, air leaks from the staple line were uncommon. We conducted this retrospective study to evaluate the outcomes of patients who underwent thoracoscopic wedge resection without chest drain placement.

## Methods

### Study design

All operations were conducted at the China Medical University Hospital. Patients who underwent elective thoracoscopic wedge resection and who provided informed consent were included in this study, whereas patients who underwent mechanical pleurodesis for a pneumothorax, radical lymph node dissection for lung cancer, or bilateral lung surgery at the same time were excluded. Between January, 2013 and July, 2015, 89 patients who underwent thoracoscopic wedge resection for metastatic pulmonary nodules (*n* = 39), infected pulmonary lesions (*n* = 18), primary lung cancer (*n* = 18), and benign pulmonary nodules (*n* = 14) were enrolled in this study. No chest drain placement was considered for patients whose condition met all the following criteria: the lesion was located in the outer third of the lung field; there were no bullous or emphysematous changes in the lung; there were no air leaks, seen grossly or during a water sealing test; there was no dense adhesion of pleura; and there was no oozing or accumulation of pleural effusion. If any criteria were not met, a chest drain was placed intraoperatively. Forty-five patients whose condition did not meet the criteria were assigned to the “chest drain placement group” (group A), and 44 patients whose condition met the criteria were assigned to the “no chest drain placement group” (group B). Patient characteristics, specimen data, and postoperative conditions were analyzed and compared between the groups.

### Operative management

**Fig. 1:**
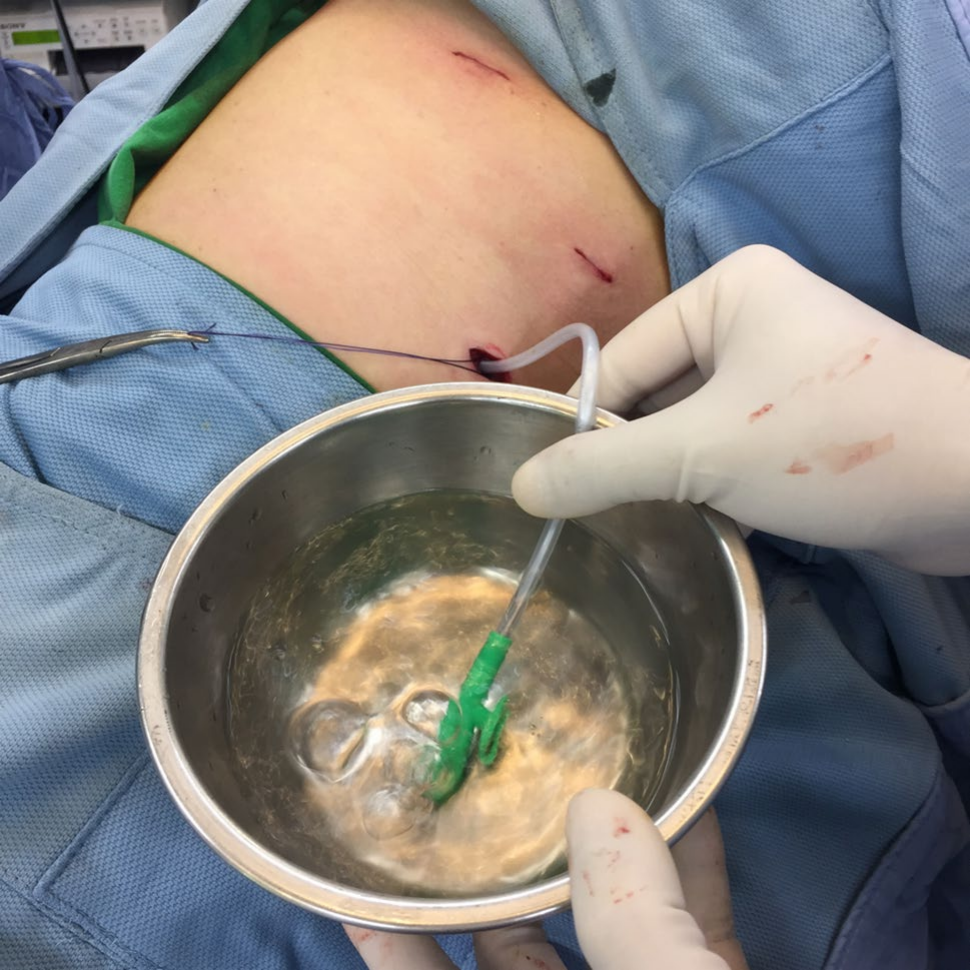
In group B (no chest drain), air evacuation was achieved by temporarily inserting a nasogastric (NG) tube into the pleural cavity with a bowl of aseptic water as the water seal system. The NG tube was removed when there was no air leak during lung inflation

All patients underwent thoracoscopic wedge resection of the lung via the 4th, 6th, and 8th intercostal spaces, using the three ports technique with 10 mm ports, under general anesthesia with single lung ventilation. A lung parenchyma resection was performed with ECHELON FLEX™ ENDOPATH^®^ Staplers (3.5/4.1 mm staple height, Ethicon, US). In group A, a #16 French SKATER drain (Angiotech, USA) was placed via the 6th intercostal port. In group B, air evacuation was achieved by temporarily inserting a nasogastric (NG) tube into the pleural cavity, with a bowl of aseptic water as the water seal system (Fig. 1). When no air leaked while the lung was inflated (maximum airway pressure of 25 mm Hg), the NG tube was removed, and the residual wound was immediately closed with subcutaneous sutures.

### Postoperative management

Patients were not allowed any oral intake for 4 h postoperatively, after which oral pain control was given, as acetaminophen 500 mg, four times a day. An intramuscular opioid (meperidine 30 mg, with a minimum 6 h interval) was provided for patients with intolerable pain. Chest radiographs were arranged just after surgery. If there were no abnormal findings or clinical symptoms, additional chest radiographs were obtained on postoperative days 2 and 4, and then on the day of discharge for both groups. The chest drain was removed when the amount of drainage was <100 ml/day and there was no evidence of ongoing bleeding or air leakage. Patients were not discharged until at least 6 h after drain removal.

### Statistical analysis

All continuous variables were compared with the Student’s *t* test and categorical variables were compared with the Fisher’s exact test. All probability values were two tailed, and *p* < 0.05 was considered significant. Statistical analysis was performed using Stata 13.1 software (StataCorp, USA).

### Ethical statement

This retrospective study was approved by the research ethics committee of China Medical University & Hospital, Taichung, Taiwan (CMUH104-REC1-119).

## Results

### Patient characteristics

Table 1 lists the patient characteristics. There was no significant difference between the groups in terms of age, gender, and forced expiratory volume in one second (FEV1)/forced vital capacity (FVC). However, the group A patients had significantly poorer FEV1 values (84.36 ± 18.05 vs. 95.87 ± 13.38 %, *p* = 0.002) and significantly fewer normal spirometric results (42.86 vs. 75.00 %, *p* = 0.004) than the group B patients. Four patients in group A and three patients in group B did not undergo a preoperative lung function test. Six patients in group A could not complete the preoperative lung function test and were considered to have abnormal lung function.

**Table 1:** Patient characteristics

	Group A (*N* = 45)	Group B (*N* = 44)	*p* value
Gender			
Female	24	23	
Male	21	21	1.000
Age (years)	56.98 ± 16.33	54.14 ± 12.94	0.366
FEV1/FVC (%)	73.22 ± 2.95	74.22 ± 4.13	0.225
FEV1 (%)	84.36 ± 18.05	95.87 ± 13.38	0.002
Spirometry			
Normal	18	30	
Abnormal	24	10	0.004

Group A: patients with chest drain placement; Group B: patients without chest drain placement

*FEV1* forced expiratory volume in one second, *FVC* forced vital capacity

### Specimen data

Table 2 lists the specimen data. There was no difference between group A and group B in terms of the number of wedge resections and final pathology reports. However, the group A patients had a significantly higher resected lung volume (59.02 ± 52.84 vs. 17.81 ± 14.67 cm^3^, *p* < 0.001), significantly greater maximum tumor-pleura distance (6.46 ± 8.01 vs. 3.36 ± 5.36 mm, *p* = 0.034), and a significantly larger maximum tumor size (16.36 ± 10.1 vs. 9.64 ± 5.76 mm, *p* < 0.001) than the group B patients. The variable “maximum tumor-pleura distance” was defined as the maximum distance measured from the tumor edge to the nearest visceral pleura or fissure for all nodules in the same patient. The variable “maximum tumor size” was defined as the maximum tumor size measured by ground glass opacity and the solid part for all nodules in the same patient. The final pathology reports are also presented in Table 2.

**Table 2:** Specimen findings

	Group A (*N* = 45)	Group B (*N* = 44)	*p* value
Resected lung volume (cm^3^)	59.02 ± 52.84	17.81 ± 14.67	<0.001
Maximum tumor-pleura distance (mm)	6.46 ± 8.01	3.36 ± 5.34	0.034
Maximum tumor size (mm)	16.36 ± 10.1	9.64 ± 5.76	<0.001
Wedge number	1.42 ± 0.69	1.2 ± 0.46	0.837
Final pathology			
Infectious lesion	9	9	
Benign nodule	7	7	
Metastatic lesion	19	20	
Primary lung cancer	10	8	

Group A: patients with chest drain placement; Group B: patients without chest drain placement

### Postoperative conditions

Table 3 summarizes the postoperative data. The group A patients had a significantly longer postoperative hospital stay than the group B patients (4.13 ± 0.87 vs. 3.14 ± 0.98 days, *p* < 0.001). However, there was no difference between the groups in the number of meperidine injections and postoperative subcutaneous emphysema. Regular follow-up chest radiographs confirmed the absence of a postoperative pneumothorax in all patients from both groups.

**Table 3:** Postoperative conditions

	Group A (*N* = 45)	Group B (*N* = 44)	*p* value
Postoperative hospital stay (days)	4.13 ± 0.87	3.14 ± 0.98	<0.001
Number of meperidine injection (time)	1.44 ± 1.14	0.98 ± 1.21	0.064
Postoperative subcutaneous emphysema			
Yes	24	15	
No	21	29	0.088
Postoperative pneumothorax			
Yes	0	0	
No	45	44	1.000

Group A: patients with chest drain placement; Group B: patients without chest drain placement

## Discussion

Minimally invasive thoracoscopic techniques, which aim to decrease the wound size, reduce postoperative pain, and preserve as much lung function as possible, are being used widely in many types of thoracic surgery. The pain caused by chest tube placement has become significant [7] and previous reports have studied the consequences of early timed chest tube removal [8, 9]. Some reports have discussed the selection criteria for patients who did not require chest tube placement following thoracoscopic lung biopsy or even wedge resection [10, 11]. We used these criteria for selecting patients in this study. In our experience, the decision regarding chest tube placement was also dependent on the resected lung volume, tumor size, distance from the tumor to pleura, and the number of wedge resections [6].

The major source of air leaks intraoperatively included the following: a raw surface after pneumolysis, the staple line after parenchyma resection, and bullae rupture caused by positive pressure ventilation. Air leaks from the staple line will be less common if the surgeon chooses the appropriate staple for parenchyma resection [12]. Thus, intraoperative lung adhesion, emphysematous change, or bullae formation are considered to be the most important factors for predicting postoperative air leaks. There was no air leak lasting more than 24 h postoperatively in our group A patients. This study excluded pneumothorax patients who routinely received mechanical pleurodesis by performing pleural abrasion during surgery to prevent recurrence, as these patients would probably need chest tube placement because of the raw surface and oozing of injured pleura.

According to our retrospective data review of all 89 patients (group A and B), there was no severe postoperative pneumothorax or subcutaneous emphysema for which reinsertion of a chest tube would have been indicated. There was only minor, nonprogressive subcutaneous emphysema in 21 group A patients and 29 Group B patients. There were no mortalities during hospitalization or in the 30 days of outpatient follow-up. Postoperative pain was evaluated by comparing the number of meperidine injections needed between the two groups, although the results were controversial. This could be because we used a #16 French drain, which is thinner than traditional chest tubes and may reduce postoperative pain. The mean duration of chest drain placement was 2.2 days in group A. There was a significant difference in the length of the postoperative hospital stay between the two groups. A shorter hospital stay promotes patients resuming their daily lives and saves on medical costs.

### Study limitations

This study had several limitations. First, as these 89 patients were reviewed retrospectively, some data may not have been collected correctly or appropriately. Second, a pulmonary function test could only be obtained preoperatively; however, several patients could not complete the testing. Furthermore, postoperative pulmonary function may be an important factor when evaluating a patients’ recovery. Third, the severity of any emphysema changes and bullae formation in the lung could only be assessed by chest computerized tomography and gross observation during the operation. There was no suitable quantification for parenchyma changes, and the surgeon’s decisions during the operation could only be estimated based on the surgical record. Finally, although we attempted to standardize the thoracoscopic wedge resection procedure, there were variations among individuals. For example, the incision size was based on the size of the specimen. Intercostal nerve injury caused by electrocautery or instrument compression was related to the incision location and the width of the intercostal space. All these factors influence postoperative pain.

## Conclusions

There were no severe complications in patients who did not have a chest drain placed following thoracoscopic wedge resection. Furthermore, the omission of chest drain placement contributed to a shorter postoperative hospital stay. Therefore, chest drain placement following a thoracoscopic wedge resection can be avoided for selected patients who have small peripheral lesions and healthy lung parenchyma, and may be safe and beneficial.
